# Synthesis of Ultrahigh Molecular Weight Poly (Trifluoroethyl Methacrylate) Initiated by the Combination of Palladium Nanoparticles with Organic Halides

**DOI:** 10.3390/polym16192764

**Published:** 2024-09-30

**Authors:** Jian Guan, Xiaodi Yu, Minghui He, Wenfeng Han, Ying Li, Zongjian Liu, Panpan Zhang, Haodong Tang

**Affiliations:** 1 College of Chemical Engineering, Zhejiang University of Technology, Hangzhou 310014, China; guanjian2020@126.com (J.G.); yuxiaodi@zjut.edu.cn (X.Y.); 15058967256m@sina.cn (M.H.); hanwf@zjut.edu.cn (W.H.); liying@zjut.edu.cn (Y.L.); zjliu@zjut.edu.cn (Z.L.); tanghd@zjut.edu.cn (H.T.); 2 Key Laboratory of Chemical and Biological Processing Technology for Farm Products of Zhejiang Province, Zhejiang Provincial Collaborative Innovation Center of AgriculturalBiological Resources Biochemical Manufacturing, School of Biological and Chemical Engineering, Zhejiang University of Science and Technology, Hangzhou 310023, China

**Keywords:** ultrahigh molecular weight, PTFEMA, Pd nanoparticles, kinetics, polymerization mechanism

## Abstract

Ultrahigh molecular weight polymers display outstanding properties and have great application potential. However, the traditional polymerization methods have inevitable disadvantages that challenge the green synthesis of ultrahigh molecular weight polymers. The paper achieved an ultrahigh molecular weight poly (trifluoroethyl methacrylate) via a novel polymerization and discussed the mechanistic, kinetic, and experimental aspects. The combination of palladium nanoparticles with ethyl 2-bromopropionate has been identified as an exceedingly efficient system for initiating the polymerization of trifluoroethyl methacrylate. An ultrahigh molecular weight poly (trifluoroethyl methacrylate) with a number-average molecular weight up to 3.03 × 10^6^ Da has been synthesized at a feeding molar ratio of [poly (trifluoroethyl methacrylate)]/[ethyl 2-bromopropionate]/[palladium nanoparticles] = 3.95 × 10^4^:756:1 at 70 °C. The reaction orders concerning palladium nanoparticles, ethyl 2-bromopropionate, and poly (trifluoroethyl methacrylate) were determined to be 0.59, 0.34, and 1.38, respectively. By analyzing a series of characterizations, we verified that the polymerization of poly (trifluoroethyl methacrylate) was initiated by the ethyl 2-bromopropionate residue radicals, which were generated from the interaction between palladium nanoparticles and ethyl 2-bromopropionate. The comparatively large size of the palladium nanoparticles provided a barrier to chain-growing radicals, promoting the synthesis of ultrahigh molecular weight polymers.

## 1. Introduction

Semifluorinated poly(meth)acrylates (SFPMAs) are known for their exceptional transparency, processability, and biocompatibility, as well as their distinct chemical and physical properties, like chemical resistance, high thermal stability, low refractive indices, and low surface energy. These characteristics render them highly promising for extensive applications, including medical facilities, optical materials, sealing materials, and coatings. Poly (trifluoroethyl methacrylate) (PTFEMA) is one of the most common SFPMAs [1,2,3,4,5,6]. Nonetheless, PTFEMA with a low molecular weight demonstrates inferior wear resistance, thermal stability, and mechanical strength, thereby restricting its potential application domains. It is widely accepted and commonly known that the molecular weight and its distribution serve as a key element in determining the mechanical qualities, processing features, and range of applications of polymer materials [7,8]. As an illustration, as the number-average molecular weight (*M*_n_) of PHFBMA varies between 265.5 kg/mol and 1300 kg/mol, the initial decomposition temperature (T_d_) is elevated from 306.4 °C to 318.7 °C, indicating that the increase in the number-average molecular weight can enhance the thermal stability of materials [9]. Therefore, the synthesis of UHMW polymers holds substantial practical significance and value.

Chen and colleagues achieved a UHMWPTFEMA with a number-average molecular weight (*M*_n_) of 1.47 × 10^6^ Da through bulk polymerization, using 0.5 wt.% 2,2′-azobis(2-methylpropionitrile) (AIBN) as an originator at 60 °C [10]. Nevertheless, the reaction time was excessively long. Through reversible deactivation radical polymerization (RDRP) technologies, the synthesis of fluorine-containing functional polymeric materials has become a hot topic in recent years [11,12,13,14,15,16]. Matyjaszewski et al. synthesized PTFEMA with a low polydispersity (Đ < 1.2) by photoinduced iron-catalyzed atom transfer radical polymerization (ATRP). This technique enables effective control of polymer chain growth [17]. Hu and coworkers synthesized the fluorine-rich polymer (21-β-CD-g-PTFEMA) with 21 arms by ATRP, in which the core was post-modified β-cyclodextrin (β-CD) and the arms were PTFEMA, and the *M*_n_ of the polymer reached 9.0 × 10^5^ Da [18]. Reversible addition-fragmentation chain transfer (RAFT) polymerization offers distinct advantages for synthesizing block copolymers with clear structures and compositions. An amphiphilic block copolymer (PMAA-*co*-PTFEMA)-*b*-PMMA-*b*-(PMAA-*co*-PTFEMA) with self-brittle functionality was efficiently synthesized via a one-pot RAFT polymerization approach [19]. However, the synthesis of UHMWPTFEMA via RDRP presents several formidable challenges, including the efficiency of the initiator, the management of reaction conditions, and the complexity of the polymerization method [20,21,22,23,24,25,26].

Recently, Lewis pair polymerization (LPP) has been formulated to efficiently synthesize SFPMAs while precisely controlling stereospecificity and molecular weight. According to Wang et al., a reliable catalyst system based on methylaluminum bis(2,6-di-*tert*-butyl-4-methylphenoxide)/1,3-di-*tert*-butylimidazolin-2-ylidene (MeAl(BHT)_2_/I^t^Bu) has been developed for the synthesis of SFPMAs. PHFBMA with a number-average molecular weight (*M*_n_) as high as 1300 kg/mol, a low polydispersity (PDI = 1.03), and PTFEMA (*M*_n_ = 919.2 kg/mol, PDI = 1.05) was prepared under mild conditions [9]. However, the high catalyst loading in the synthesis, coupled with the moisture- and air-sensitivity of the organoaluminum compound, introduces significant technical challenges for the polymerization process in the context of industrial applications.

It was discovered that through CCl_4_, Raney metals (Ni, Fe, Co) initiated the polymerization of methyl methacrylate (MMA) [27]. Otsu et al. reported that certain active metals and organic halides could trigger radical polymerization reactions of vinyl monomers. Moreover, the kind of organic halide and metal and the manner of metal preparation had a significant impact on the activity of the system in initiating polymerization reactions [28,29,30]. Yuan et al. demonstrated that metal nanoparticles, combined with organic halides, could produce ultrahigh molecular weight poly (methyl methacrylate) (UHMWPMMA) with an *M*_n_ of 4.65 × 10^6^ Da [31]. Zhang et al. claimed a novel synthesis of a UHMWPMMA with an *M*_n_ of 1.68 × 10^6^ Da was created by utilizing organosulfur compounds in tandem with transition metal carboxylates as the initiating system [32]. However, there has been little research on the polymerization and kinetics of SFPMAs with unique and intriguing characteristics, such as PTFEMA, initiated by metal nanoparticles and organic halides.

Palladium nanoparticle (Pd NP) is an effective catalyst for a lot of reactions, such as the reductive Heck reaction, Suzuki coupling, and hydrodechlorination of chlorinated organic compounds [33,34]. Traditionally, Pd nanostructures serve as a cost-effective catalyst for the catalyzed cleavage of carbon–halogen (C–X) bonds, exhibiting both a lower economic cost compared to Pt-group metals and superior catalytic activity than Cu, Ni, or Fe catalysts [35]. As reported in the literature, Pd^0^ is the active center of the reaction owing to the low electron density nature of positively charged Pd atoms [36]. Liu and colleagues have demonstrated that Pd plays a major role in the cleavage of carbon-halogen bonds catalyzed by Au@Pd bimetallic nanocatalysts. It is revealed that Au enhanced the activity of Pd atoms primarily by increasing the occupation state of Pd d-orbitals [37]. In summary, Pd NPs possess significant advantages in the activation and cleavage of carbon-halogen bonds.

Our research revealed the development of Pd NPs in conjunction with ethyl 2-bromopropionate (EBP) as an initiator that collaboratively facilitated the effective polymerization of TFEMA. Furthermore, the research explored the influence of disparate factors on the synthesis of UHMWPTFEMA while revealing the mechanism for the synthesis of UHMWPTFEMA through analytical research on the dynamics of polymerization and a series of characterizations. 

## 2. Materials and Methods

### 2.1. Materials

Trifluoroethyl methacrylate (TFEMA, 99.9%) was supplied by Leyan, China. Ethyl 2-bromoisobutyrate (EBiB, 99%), ethyl 2-bromopropionate (EBP, 99%), ethyl 2-chloropropionate (ECP, 97%), 2-bromo-2-phenylacetic acid (α-BPA, 97%), ethyl α-bromophenylacetate (α-EBP, 95%), toluene (AR), tetrahydrofuran (AR), and basic aluminum oxide (200–300 mesh) were supplied by Aladdin Inc (Shanghai, China). Palladium acetate (47% Pd) was offered by Sigma-Aldrich (St. Louis, MO, USA). 1,1-diphenyl-2-picrylhydrazyl (DPPH, 95%), n-tert-butyl-alpha-phenylnitrone (PBN, >98%), and n-dodecyl sulfide (GC, >96%) were provided by TCI (Tokyo, Japan). Inhibitors were separated from the TFEMA using columns packed with basic alumina, and the monomer purified was preserved at −20 °C.

### 2.2. Synthesis of Pd NPs

As depicted in the literature, the Pd NPs were synthesized [38]. Into a 50 mL volume of toluene, palladium(II) acetate (0.20 g, corresponding to 0.30 mmol) and n-dodecyl sulfide (1.65 g, equivalent to 4.45 mmol) were introduced. The mixture was then subjected to a heating regimen of 95 °C, sustained for 3 h. During the process, the solution color varied from orange to black, which illustrated the production of Pd nanoparticles. 

### 2.3. Synthesis of Ultrahigh Molecular Weight PTFEMA Initiated by the Combination of Pd NPs with Organic Halides 

Typically, a sealed reaction vessel containing the magnetic stirring bar was evacuated in a high vacuum and refilled with nitrogen gas. Having previously been purified using nitrogen, TFEMA was introduced into the reaction vessel and then integrated with organic halides and Pd NPs using a nitrogen-purged syringe. Subsequently, the vessel was placed into an oil bath preheated to a specified temperature. At disparate time intervals, aliquots of the reaction mixture were extracted using a syringe fitted with one long needle and were immediately stored in a refrigerator. These samples were later analyzed for monomer conversion and size exclusion chromatography (SEC).

### 2.4. Characterization

Monomer conversion was determined through a gravimetric method. The molecular weight and polydispersity index (PDI) of the polymers were directly construed by size exclusion chromatography (SEC) (Shimadzu Co., Kyoto, Japan) using a Waters solvent-efficient Styragel HMW-6E high-resolution column (4.6 mm × 300 mm, molecular weight range: 5.0 × 10^3^–1.0 × 10^7^ Da). Tetrahydrofuran (THF) was used as an eluent (40 °C, 0.3 mL/min). An array of polystyrene standards were adopted to formulate the SEC calibration curve. The PDI values were calculated using the formula PDI = *M*_w_/*M*_n_, where *M*_w_ is the mass-average molecular weight and *M*_n_ is the number-average molecular weight. Furthermore, to substantiate the precision of our PDI measurements, we have performed statistical analysis on each set of PDI values.

Intrinsic viscosities [η] at 20 °C were determined using a Ubbelohde-type automatic viscosimeter (Zhuoxiang technology, Hangzhou, China) for solutions of PTFEMA in 2-methoxyethylacetate. Mean molecular weights *M*_v_ were deduced from these viscosity measurements by using the Mark-Houvink relationship:

(1) $$[\eta ]=KM_{v}^{a}$$

where the exponent *a* is near 0.77 and K is 3.4 × 10^−5^, respectively. The values of *M*_v_ for PTFEMA have been determined by Equation (1).

^1^H NMR and ^13^C NMR spectra of polymers were obtained using one Avance III 600 MHz NMR instrument (Bruker, Karlsruhe, Germany) in chloroform-d (CDCl_3_) with tetramethylsilane as the internal standard at room temperature. ^1^H NMR was used to simplify the end-group analysis of the synthesized polymers. PTFEMA with low molecular weight was prepared using the same processes as normal PTFEMA synthesis, and the reaction was purposefully stopped at a low monomer conversion rate. The low-conversion polymers were precipitated by the addition of excess n-hexane. The resulting low molecular weight PTFEMA was subsequently dried under vacuum conditions.

The synthesized PTFEMA was completely dissolved in THF, and the solution was then dropped onto a glass slide. After the complete evaporation of the solvent, the prepared samples were characterized using infrared (IR) spectroscopy. IR Affinity-1S spectrometer, manufactured by Shimadzu, was utilized. The resolution was 2 cm^−1^, the number of scans was 64, and the spectral range was 500~4000 cm^−1^.

Tecnai G2 F30 (acceleration voltage 300 kV, FEI, Hillsboro, MA, USA) was used to characterize the transmission electron microscopy (TEM). The prepared Pd NPs solution was centrifuged and dispersed into THF by adding excess methanol, sonicated for 5 min, then dripped onto an electron microscope copper grid and naturally air-dried for TEM characterization. To examine the Pd NPs after the polymerization reaction, the sample, as obtained at low conversion, was centrifuged in a high-speed centrifuge to collect the products deposited on the bottom. These products were then dispersed into THF for TEM analysis. 

X-ray photoelectron spectroscopy (XPS) was implemented on a Kratos AXIS Ultra DLD spectrometer (Thermo Kalpha, Waltham, OR, USA) and calibrated with C 1s (284.6 eV) to measure the valence state of Pd atoms in NPs (before and after polymerization). 

The radicals formed during the polymerization of TFEMA were inspected using an electron spin resonance (ESR) spectrometer (A300, Bruker Co. (Billerica, MA, USA)) with an X-band microwave at a microwave frequency of approximately 9.8 GHz and a microwave power of 20.0 mW. In the initial phase of TFEMA polymerization, PBN was added to the reaction solution as a free radical trapping agent (PBN final concentration: 0.075 mol/L) to capture the chain-propagating free radicals. A certain amount of solution was prepared in a capillary for ESR detection. 

The end group analysis on the polymer was carried out using Bruker rapifleX MALDI-TOF produced by Bruker. The matrix used was trans-2-[3-(4-tert-butylphenyl)-2-methyl-2-propenylidene] malononitrile (DCTB). DCTB was decomposed in THF at a concentration of 20.0 mg/mL. The oligomer sample was diluted to 10.0 mg/mL with THF. CF_3_COONa was dissolved in THF at a concentration of 0.5 mol/L. 10.0 μL of DCTB solution, 3.0 μL of sample solution, and 1.0 μL of CF_3_COONa solution were mixed. After mixing evenly, 0.5 μL of the mixed solution fell on the plate and was tested under cationic reflection or linear mode conditions.

A series of polymer solutions were prepared in THF to explore the influence of molecular weight on contact angle. The solution was fully coated on a clean slide and dried for 48 h to form a film. The contact angle of the polymer film to water was measured at room temperature by OCA20 (Data-Physics Instruments GmbH, Filderstadt, Germany) with a droplet volume of 2.0 μL and was tested at least six times.

## 3. Results and Discussion

### 3.1. Synthesis of Ultrahigh Molecular Weight PTFEMA Using Organic Halides Combined with Pd NPs as Initiators

The Pd NPs were integrated with various organic halides, including EBP, EBiB, and ECP, and were screened as initiators to initiate bulk polymerization of TFEMA (Table 1). Evidently, the Pd NPs alone could not initiate polymerization in the absence of any organic halides in the reaction system (entry 1). The polymerization rate is slowly initiated by α-EBP, α-BPA, and ECP (entry 2–4). Compared with the above-mentioned organic halides, EBiB and EBP accelerated the polymerization rate of TFEMA and generated UHMW polymers (entry 5, 6). Correspondingly, as the mass-average molecular weight of the polymer increases, so does the viscosity of the polymer solution, as well as the viscosity-average molecular weight [39]. Especially, a UHMWPTFEMA with a number-average molecular weight up to 3.03 × 10^6^ Da was obtained at [TFEMA]/[EBP]/[Pd NPs] = 3.95 × 10^4^:756:1, as measured by SEC (Figure S1). Therefore, the TFEMA/EBP/Pd NPs system was selected to prepare a UHMWPTFEMA due to the EBP showing the highest molecular weight.

### 3.2. Synthesis of Ultrahigh Molecular Weight PTFEMA Using EBP as an Initiator Combined with Pd NPs

Apart from different initiators, the molecular weight and polydispersity of PTFEMA were influenced by various factors, including temperature, initiator concentration, and Pd NPs concentration. The complex interaction of these factors has always been a challenge for precisely controlling molecular weight and polydispersity. Consequently, we investigated the influences of discrepant reaction parameters on the molecular weight and MWD of PTFEMA.

As shown in Table 2, the conversion decreased due to a decrease in Pd NPs concentration, but the molecular weight increased (entry 1, Table 2). Additionally, it was observed that a reduced concentration of EBP led to diminished monomer conversion. However, the molecular weight of PTFEMA tended to rise and subsequently decline (entry 2, Table 2). Furthermore, we have noted that an increase in the PDI leads to a decrease in the value of *M*_v_/*M*_w_. This is primarily attributed to the fact that the lower molecular weight polymers within the MWD have a more significant impact on viscosity than the higher molecular weight polymers [39]. The rise in Pd NPs concentration would provide more active sites for the polymerization reaction, while the rise in initiator concentration would increase the number of free radicals. Both could raise the polymerization reaction rate. However, the number of polymer chains produced would increase with more active sites and free radicals. At the same monomer conversion, as polymer chains increased, the proportion of short-chain molecules increased and that of long-chain molecules decreased, resulting in a decline in the molecular weight [20,40,41]. The molecular weight of PTFEMA, determined at a molar TFEMA/EBP/Pd NPs ratio of 3.95 × 10^4^:298:1.5, was observed to be lower than that at a molar ratio of 3.95 × 10^4^:527:1.5, indicating that the synthesis of UHMWPTFEMA may not be feasible under conditions of excessively low initiator concentration (entry 2, Table 2). Further increasing the temperature was found to augment the polymerization rate but resulted in a minor reduction of the molecular weight of PTFEMA (entry 3, Table 2). Moreover, the MWD of the polymer is narrow. Since activation energy follows the sequence of chain transfer > propagation > termination, raising the temperature boosts the chain transfer coefficient (k_tr_) more than the chain propagation coefficient (k_p_) and chain termination rate coefficient (k_t_). The chain transfer reaction in polymerization typically results in a reduction in the molecular weight of polymers [42].

### 3.3. Kinetics of the Polymerization of TFEMA Initiated by the Combination of Pd NPs with EBP

**Figure 1:**
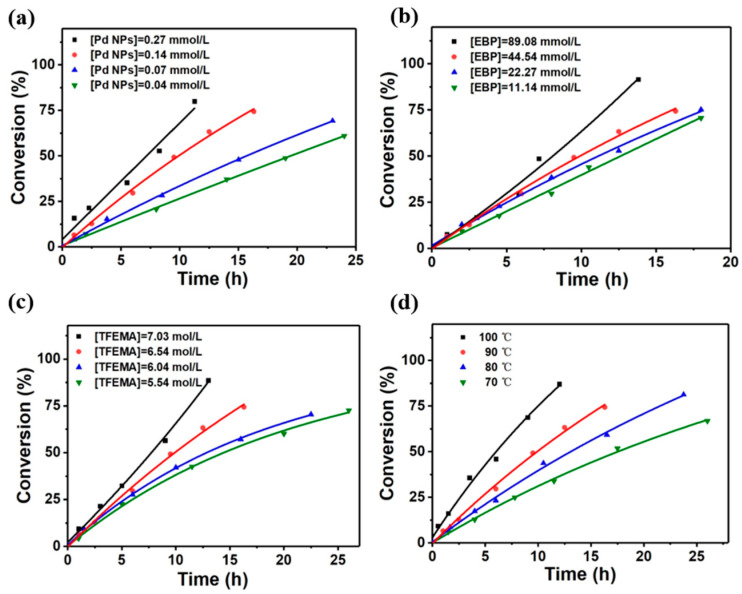
Conversion-time plots of the polymerization of TFEMA at different Pd NPs concentrations (**a**), EBP concentrations (**b**), monomer concentrations (**c**), and different temperatures (**d**).

The study of the kinetics of polymerization helps explore the reaction mechanism in theory and for controlling the process conditions in actual production. The experimental conversion versus time curve typically represents the reaction rate, as shown in Figure 1.

Obviously, the polymerization reaction rate increased significantly as the concentration of EBP, Pd NPs, and TFEMA increased, which was also facilitated by the increase in reaction temperature (Figure 1a–d). The study of polymerization reaction kinetics was usually carried out in the early phase of polymerization when the conversion reached 10% or less. The initial reaction rate (−dc/dt) was obtained from the conversion-time curve of the polymerization reaction. Subsequently, the plot of ln(−dc/dt) versus ln(c) should be linear, with the slope equal to the reaction order, whereby c is the concentration of reactants. As a result, it was shown that the reaction orders for the concentrations of Pd NPs, EBP, and TFEMA were 0.59, 0.34, and 1.38 at 90 °C, respectively. Consequently, the polymerization kinetic equation was denoted as R = −dc/dt ∝ [TFEMA]^1.38^ [Pd]^0.59^ [EBP]^0.34^. The reaction orders of the polymerization of TFEMA induced by the integration of EBP and Pd NPs differed from the reaction orders of the initiator and monomer in a typical free radical polymerization, which reached 0.5 and 1, separately. This indicates that the two processes have different kinetic mechanisms. The activation energy of the polymerization was figured out to be 42.5 kJ/mol by linearization of the Arrhenius equation.

### 3.4. Preliminary Investigation of the Mechanism of TFEMA Polymerization Initiated by the Combination of Pd NPs with EBP

#### 3.4.1. TEM Images and XPS Analysis of the Pd NPs before and after Polymerization 

**Figure 2:**
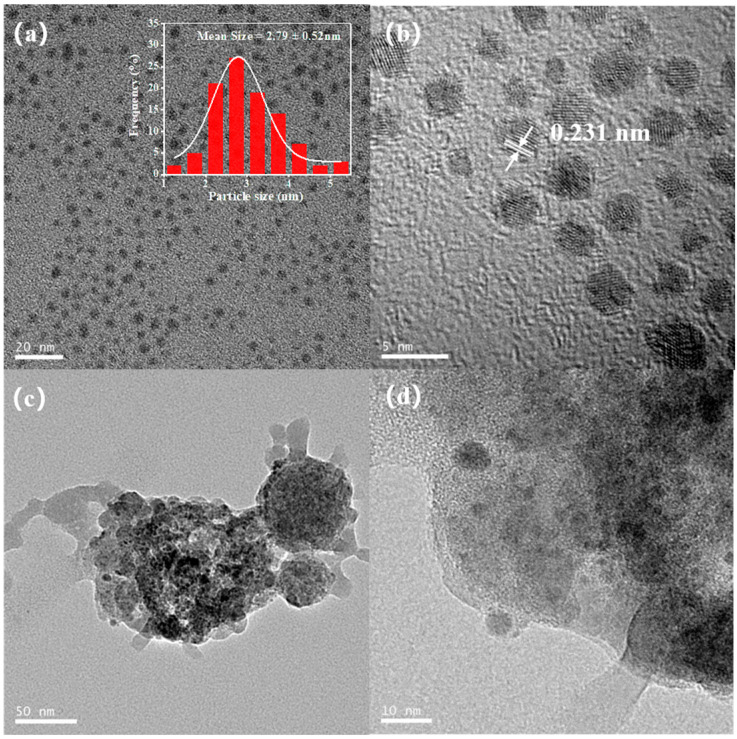
TEM image of Pd NPs (**a**) and HRTEM image of Pd NPs (**b**); TEM of Pd NPs collected at the initial stage of polymerization (**c**,**d**).

Figure 2a presents a low-resolution image of the Pd NPs. The Pd NPs spread narrowly, with an average diameter of 2.79 ± 0.52 nm. The lattice distance of synthesized Pd NPs reached 0.231 nm by high-resolution TEM imaging (Figure 2b), which was consistent with the (111) plane of the palladium crystal lattice [42]. Figure 2c clearly reveals the presence of an amorphous polymer-like deposit on the surface of Pd NPs. This finding implied that the polymerization process might take place on the surface of the Pd NPs or their immediate vicinity. As shown in Figure 2d, a certain degree of aggregation of collected Pd NPs is observed, attributed to Van der Waals interactions. 

**Figure 3:**
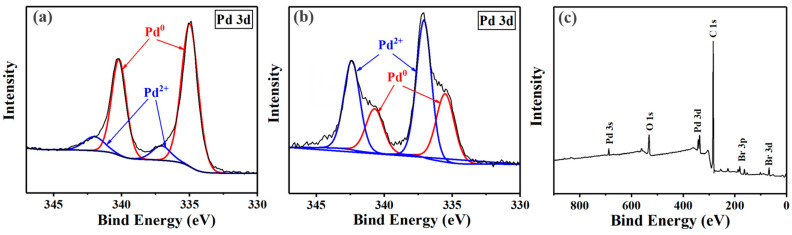
XPS spectra of Pd NPs (**a**); XPS spectra and wide-scan XPS spectra of Pd NPs collected at the initial stage of polymerization (**b**,**c**).

The electron transfer process from Pd NPs to the C-Br bond of EBP could be confirmed by the XPS spectra in Figure 3. In the XPS spectra of the Pd 3d area, the peaks at 342.1 and 337.0 eV could be assigned to the Pd^2+^ species, while the peaks at 340.2 and 335.0 eV could be assigned to the Pd^0^ species [43,44,45]. The content of Pd^2+^ estimated from the XPS spectra increased from ~12.7% to 63.3%, indicating the creation of Pd^2+^-containing species. As illustrated in Figure 3c, the characteristic peaks corresponding to bromine (Br) were discernible. This observation suggested that the electron transfer process included the transfer of a Br atom from EBP to Pd NPs. Consequently, it led to the creation of PdBr_2_ on the surface of the Pd NPs. Correspondingly, this transfer resulted in the formation of a radical residue from EBP, which initiated the polymerization of TFEMA. 

#### 3.4.2. Characterization of PTFEMA: Infrared and Carbon Nuclear Magnetic Resonance Spectroscopy Analysis

**Figure 4:**
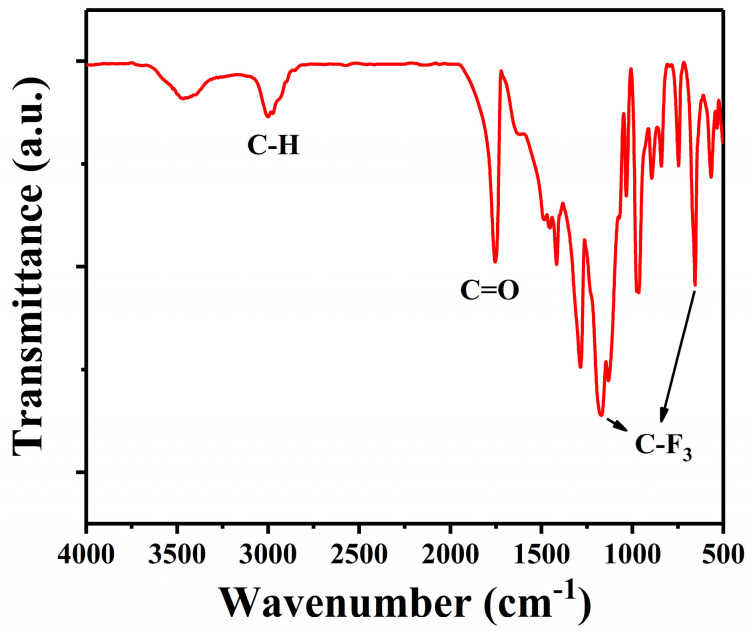
FT−IR spectra of PTFEMA initiated by EBP with Pd NPs.

The IR spectrum of PTFEMA provided valuable insights into the molecular vibrations and functional groups present in the polymer. As shown in Figure 4, the peak at 2980 cm^−1^ corresponded to a saturated C–H stretching vibration peak, and the strong peak of 1753 cm^−1^ was assigned to the stretching vibration of C=O groups. In addition, the bands at 1185 and 655 cm^−1^ were attributed to the stretching and bending vibration of the C–F groups, respectively. In addition, the characteristic stretching of the TFEMA bond at 1649 cm^−1^ disappeared, which was attributed to the stretching vibration of the C=C group, indicating a successful polymerization and purification process that has effectively removed any residual monomers [46,47].

**Figure 5:**
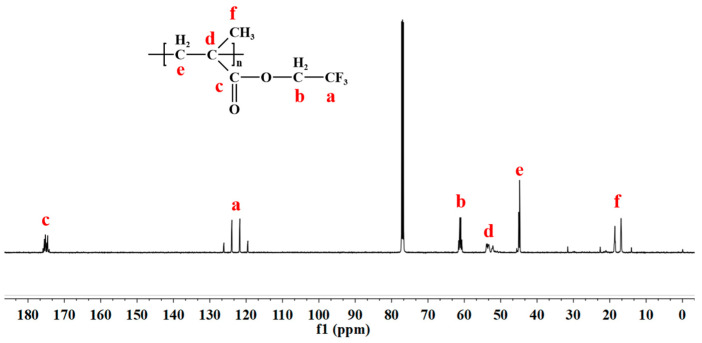
^13^C NMR spectrum of the prepared PTFEMA.

In the ^13^C NMR spectrum of PTFEMA (Figure 5), peaks attributed to the six kinds of carbon atoms in PTFEMA are well assigned. The peak c at 175 ppm was attributed to the carbonyl carbon (C=O) in the ester group of PTFEMA. A characteristic quartet of peaks centered around 125 ppm was observed, corresponding to the carbon atoms of the –CF_3_ group, with the splitting attributed to the spin-spin coupling effect of the adjacent fluorine atoms. Peaks d and e were assigned to the methylene carbon adjacent to the carbonyl group and the methylene carbon in the main chain, respectively. Additionally, peak b around 60 ppm was identified as the methyl carbon in the trifluoroethyl side chain, further substantiating the presence of this functional group. The double splitting peaks f were assigned to –CH_3_, attributed to the head-head and head-tail combination of TFEMA in the polymer chain, respectively [46,48].

#### 3.4.3. End-Group Analysis of the Prepared Low Molecular Weight PTFEMA

**Figure 6:**
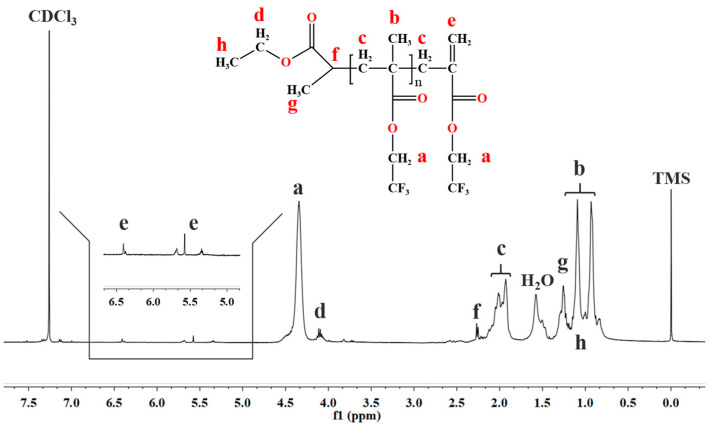
^1^H NMR spectrum of the prepared low molecular weight PTFEMA.

In terms of mechanism research on polymerization, it was crucial to examine the structures of polymer chains and identify the end groups of polymers, with a low number-average molecular weight PTFEMA (*M*_n_ = 2657 Da, obtained from SEC) prepared and the ^1^H NMR spectrum exhibited in Figure 6. As described in the literature [49,50,51,52], peaks a, b, and c could be assigned to the methylene (–OCH_2_–CF_3_), methyl (–CH_3_), and methylene groups (–CH_2_–), respectively. An overlap of methyl groups h and g of EBP with methyl group b of PTFEMA was noted. In addition, we observed a tiny signal peak d, which is a resonance peak due to the chemical shift of the methyl group in EBP (–OOCH_2_CH_3_). This finding indicated that the PTFEMA macromolecular chain was end-capped with EBP residue and that the number-average molecular weight (*M*_n_) of PTFEMA was thus determined as 2800 Da based on the combination of peak a and a neighboring peak d. This value was in close agreement with the *M*_n_ measured by the SEC. Based on the analysis in references [53,54,55], we clarified that the peaks e, which appear around 6.4 and 5.6 ppm, correspond to the olefinic protons of the carbon-carbon double bond resulting from the chain termination of PTFEMA. The signal at 5.6 ppm splits into two peaks due to the tacticity of the terminal diad. This observation suggests that the PTFEMA also had a terminal vinylidene structure. 

**Figure 7:**
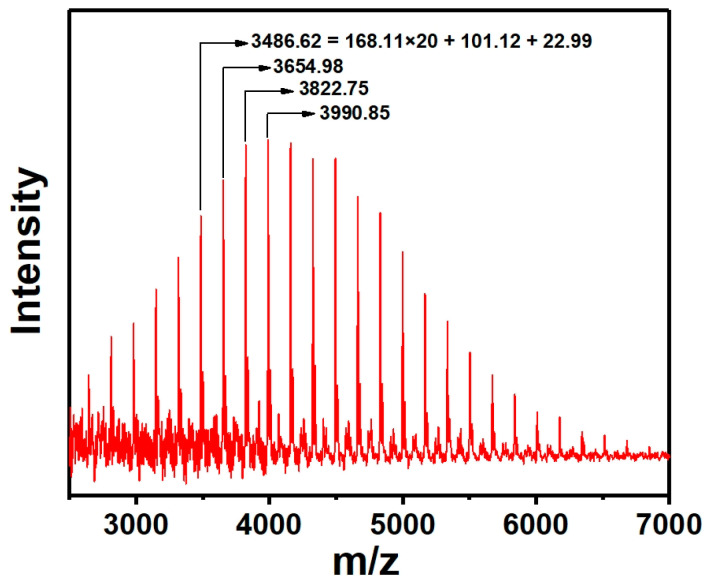
MALDI−TOF result of the prepared low molecular weight PTFEMA.

To further verify the structural information of the polymer, an end-group structure analysis of PTFEMA was conducted using MALDI−TOF, as shown in Figure 7. The signal values are 3486.62, 3654.98, 3822.75, and a range of peaks with an interval of 168. This interval aligned precisely with the molecular weight of the PTFEMA structural unit. The peak at 3486.62 was subjected to further analysis and was found to be exactly the sum of the molecular weights of twenty PTFEMA structural units, initiator residues, and Na^+^. According to the above results, the low molecular weight PTFEMA has an initiator residue with a terminal group, which matches the structural formula shown in Figure 6.

#### 3.4.4. ESR Analysis for the Polymerization Initiated by EBP with Pd NPs

As proposed in our previous work [31,32], the polymerization of TFEMA triggered by EBP with Pd NPs might also follow a radical mechanism. 1,1-Diphenyl-2-picrylhydrazyl (DPPH) is recognized as a free radical scavenger. We initially used DPPH for a preliminary assessment of the active intermediates involved in the polymerization process. The impact of DPPH, when introduced as a free radical trapping agent at a specific concentration into the polymerization system, was evaluated. The findings from this experiment are detailed in Table 3. 

**Figure 8:**
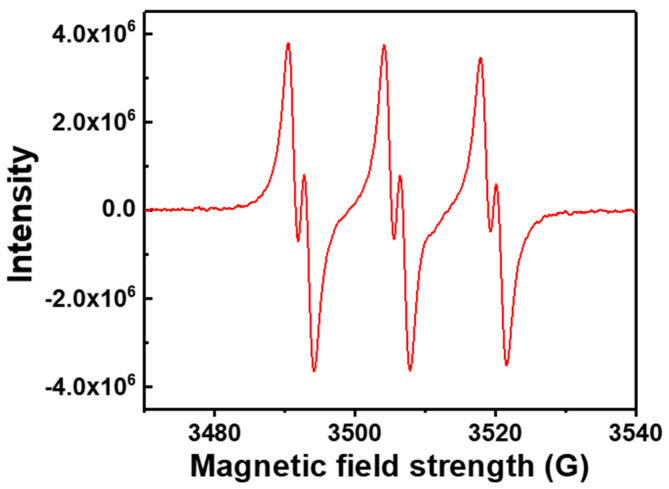
ESR spectrum from the polymerization of TFEMA initiated by EBP with Pd NPs.

The polymerization process was entirely impeded by the addition of DPPH at a concentration of 3.2 × 10^−3^ mol/L. It can thus be concluded that the polymerization of TFEMA belongs to radical polymerization. As seen in Figure 8, the ESR approach was therefore used to identify radical intermediates in the polymerization. A robust triple doublet signal was observed, which provided evidence that the reactive species of polymerization are carbon radicals. The splitting of ESR signals is due to the hyperfine coupling of carbon radicals to the ^14^N nucleus and β-proton of PBN [31,32,56,57,58,59]. Consequently, it is proven that the polymerization of TFEMA initiated by EBP with Pd NPs in fact follows a radical mechanism. 

#### 3.4.5. Mechanism Model for the Polymerization of PTFEMA Initiated by EBP with Pd NPs

**Figure 9:**
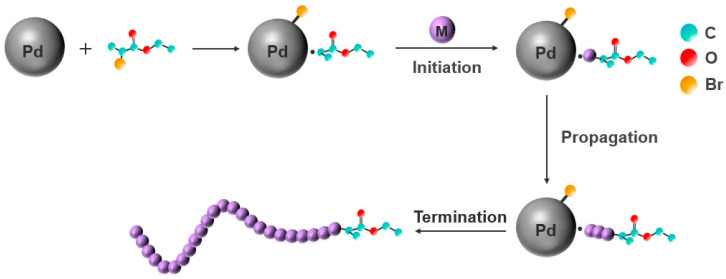
Mechanism model for the polymerization of PTFEMA initiated by EBP with Pd NPs.

Based on the above experimental and characterization results, a potential mechanism model was proposed, as illustrated in Figure 9. Firstly, EBP was adsorbed on the Pd NPs surfaces. It follows that carbon radicals close to the Pd NPs had emerged due to an electron transfer from the Pd NPs to the C-Br bond. Subsequently, the radicals initiated the TFEMA monomer, which underwent rapid chain propagation near the Pd NPs. Lastly, the polymerization reaction was terminated through a disproportionation reaction. As the reaction largely emerged on the surface of Pd NPs, the chain propagating radicals were safeguarded due to steric hindrance between Pd NPs. In consequence, the EBP/Pd NPs system created a favorable condition for preparing UHMWPTFEMA.

### 3.5. Hydrophobicity Test of PTFEMA Films with Different Molecular Weights

**Figure 10:**
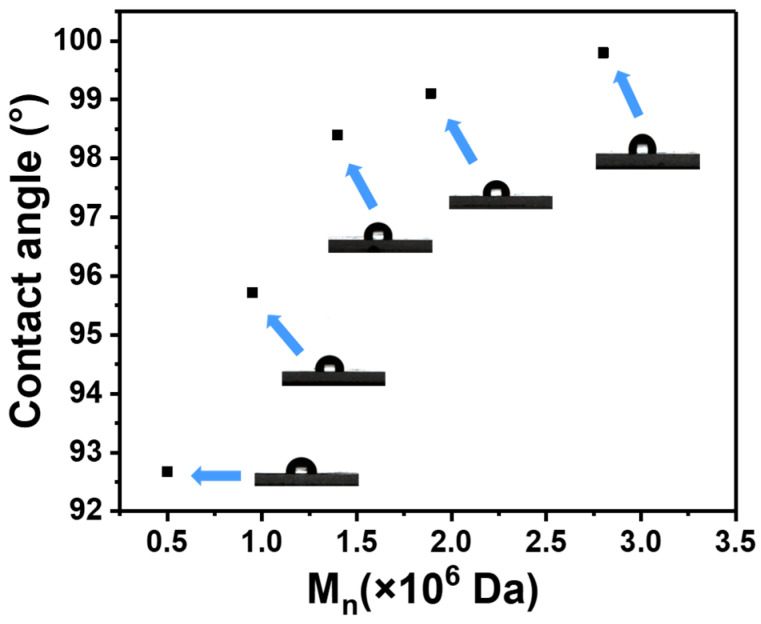
Evolution of the contact angle of PTFEMA films vs. the molecular weight of PTFEMA.

Figure 10 demonstrates that the water contact angles on the surfaces of PTFEMA films relate to the number-average molecular weight (*M*_n_) of PTFEMA. The literature suggests that this phenomenon is chiefly ascribed to the fact that the surface roughness of the PTFEMA film increases with the *M*_n_ of PTFEMA [10]. An increase in the *M*_n_ of PTFEMA corresponds to a higher degree of polymer chain entanglement. This significantly impacts the mobility of the PTFEMA chains during the film-forming process, resulting in a rougher surface. This increased roughness augments the surface of the solid, thereby geometrically enhancing the hydrophobicity by the Wenzel model [60,61]. Consequently, UHMWPTFEMA presents substantial potential for the development of superhydrophobic materials.

**Figure 11:**
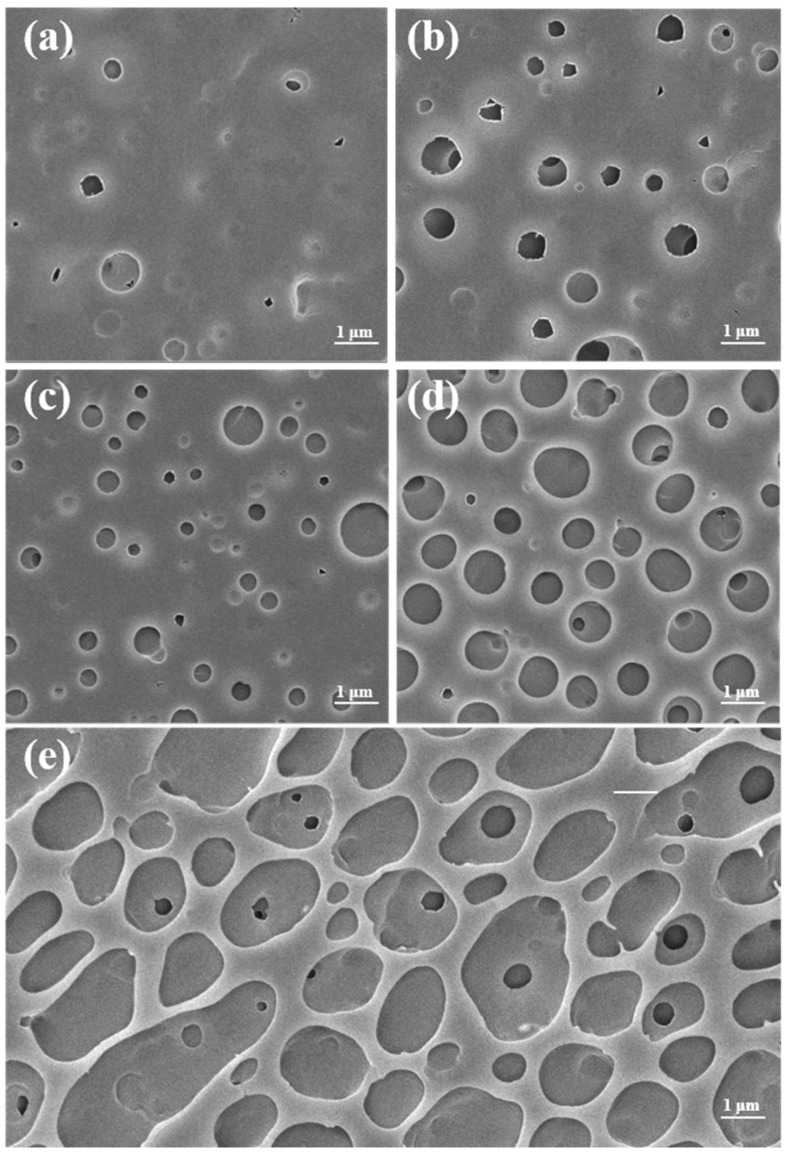
SEM images of the surfaces modified with PTFEMA of different molecular weights. (**a**) *M*_n_ = 5.06 × 10^5^ Da, (**b**) *M*_n_ = 9.50 × 10^5^ Da, (**c**) *M*_n_ = 1.42 × 10^6^ Da, (**d**) *M*_n_ = 1.89 × 10^6^ Da, (**e**) *M*_n_ = 2.80 × 10^6^ Da.

In order to better confirm the relationship between the molecular weight of PTFEMA and hydrophobicity, we have studied the morphology of the polymer films by SEM (Figure 11). SEM images reveal a transition from a relatively smooth surface at lower molecular weight to a rougher, more porous surface as the molecular weight increases. The formation of these pores could be attributed to the “breath figures”. The effect known as the “breath figures”, a phenomenon where water vapor in the air condenses within a polymer solution and leads to the formation of cellular structures on the surface of a polymer film, becomes more pronounced with higher molecular weights [10,62]. The hydrophobicity of the films featuring a honeycomb-like structure is anticipated to be significantly enhanced because of the reduction of contact areas between the water droplets and the films [63].

## 4. Conclusions

Briefly, our research put forward a new pathway to the synthesis of UHMWPTFEMA using EBP as an initiator with Pd NPs. Under the conditions of a molar proportion of [TFEMA]/[EBiB]/[Pd NPs] = 3.95 × 10^4^:756:1 and a reaction temperature of 70 °C, a UHMWPTFEMA with a number-average molecular weight as high as 3.03 × 10^6^ Da was synthesized. The reaction orders concerning Pd NPs, EBP, and TFEMA were calculated to be 0.59, 0.34, and 1.38, separately. It was verified that the polymerization reaction was initiated by free radicals from the initiator residue, and the generation of these free radicals involved an interaction between Pd NPs and the EBP. Furthermore, the nanoparticles, due to their inherent adsorption properties and giant size as physical barriers, reduced the probability of bimolecular terminations. Given the broad applications of fluorinated materials, the strategy of Pd NPs with organic halides offers a novel way for the synthesis of fluorinated UHMW polymers.

## Figures and Tables

**Table 1:** Bulk polymerizations of TFEMA initiated by organic halides in the presence of Pd NPs ^1^.

Entry	Initiator ^2^	TFEMA/Init./Pd NPs (Molar Ratio)	Conv. (%)	*M*_n_ (Da)	*M*_w_ (Da)	PDI (95% CI)	η (dL/g)	*M_v_* (Da)
1	-	3.95 × 10^4^:0:1	-	-	-	-	-	-
2	α-EBP	3.95 × 10^4^:756:1	16.9	5.81 × 10^5^	1.71 × 10^6^	2.87 ± 0.07	1.26	1.98 × 10^6^
3	α-BPA	3.95 × 10^4^:756:1	5.6	4.75 × 10^5^	1.11 × 10^6^	2.35 ± 0.04	1.11	1.24 × 10^6^
4	ECP	3.95 × 10^4^:756:1	12.4	5.10 × 10^5^	2.84 × 10^6^	5.42 ± 0.41	1.59	3.63 × 10^6^
5	EBiB	3.95 × 10^4^:756:1	46.7	2.55 × 10^6^	1.06 × 10^7^	4.53 ± 0.19	3.88	1.47 × 10^7^
6	EBP	3.95 × 10^4^:756:1	43.0	3.03 × 10^6^	1.54 × 10^7^	4.99 ± 0.07	4.00	1.53 × 10^7^

^1^ All polymerization reactions were conducted at 70 °C under the protection of nitrogen for 22 h. ^2^ Chemical structures of the substances α-EBP, α-BPA, ECP, EBiB, and EBP were shown in Figure S2.

**Table 2:** Polymerization of TFEMA initiated by EBP in the presence of Pd NPs.

Entry	TFEMA/Init./Pd NPs (Molar Ratio)	T (°C)	Time (h)	Conv. (%)	*M*_n_ (Da)	*M*_w_ (Da)	PDI (95% CI)	η (dL/g)	*M_v_* (Da)
1	3.95 × 10^4^:756:2	80	18	98.7	1.30 × 10^6^	6.37 × 10^6^	4.97 ± 0.08	2.43	7.64 × 10^6^
	3.95 × 10^4^:756:1.5	80	20	95.8	2.19 × 10^6^	1.11 × 10^7^	4.85 ± 0.21	3.69	1.38 × 10^7^
	3.95 × 10^4^:756:1	80	20	65.8	2.51 × 10^6^	1.23 × 10^7^	4.62 ± 0.19	3.76	1.41 × 10^7^
2	3.95 × 10^4^:756:1.5	80	20	95.8	2.19 × 10^6^	1.11 × 10^7^	4.85 ± 0.21	3.69	1.38 × 10^7^
	3.95 × 10^4^:527:1.5	80	20	81.2	2.36 × 10^6^	1.36 × 10^7^	4.74 ± 0.21	4.25	1.65 × 10^7^
	3.95 × 10^4^:298:1.5	80	20	58.3	1.32 × 10^6^	9.50 × 10^6^	8.37 ± 0.54	3.02	1.05 × 10^7^
	3.95 × 10^4^:69:1.5	80	20	49.8	1.17 × 10^6^	9.66 × 10^6^	6.55 ± 0.37	3.15	1.11 × 10^7^
3	3.95 × 10^4^:527:1.5	70	24	76.7	2.48 × 10^6^	1.35 × 10^7^	5.70 ± 0.23	3.86	1.46 × 10^7^
	3.95 × 10^4^:527:1.5	90	13	82.6	1.72 × 10^6^	7.08 × 10^6^	4.14 ± 0.06	3.55	1.31 × 10^7^
	3.95 × 10^4^:527:1.5	100	9	90.3	1.61 × 10^6^	5.47 × 10^6^	3.25 ± 0.07	3.34	1.21 × 10^7^

**Table 3:** Effect of DPPH on the polymerization of TFEMA initiated by EBP with Pd NPs.

Entry	TFEMA/Init./Pd NPs (Molar Ratio)	DPPH (mol/L)	Time (h)	T (°C)	Conv. (%)
1	3.95 × 10^4^:756:1	0	17	90	81.1
2	3.95 × 10^4^:756:1	3.2 × 10^−3^	17	90	-

## Data Availability

Data are contained within the article.

## Supplementary Materials

The following supporting information can be downloaded at: https://www.mdpi.com/article/10.3390/polym16192764/s1, Figure S1: SEC curve of the ultrahigh molecular weight PTFEMA. Figure S2: Chemical structures of the substances α-EBP, α-BPA, ECP, EBiB and EBP (Table 1).
