# Supplementary material for: Synthesis of Ultrahigh Molecular Weight Poly (Trifluoroethyl Methacrylate) Initiated by the Combination of Palladium Nanoparticles with Organic Halides
Source: Polymers (Basel). 2024 Sep 30;16(19):2764. doi: 10.3390/polym16192764 (PMC11479292; doi:10.3390/polym16192764)
Supplement: Supplementary file 1 [file polymers-16-02764-s001.zip › polymers-3195656-supplementary.pdf]

Supplementary Information

Synthesis of Ultrahigh Molecular Weight Poly (Trifluoroethyl Methacrylate) Initiated by the Combination of Palladium Nanoparticles with Organic Halides

Jian Guan [1], Xiaodi Yu [1], Minghui He [1], Wenfeng Han [1], Ying Li [1], Zongjian Liu [1], Panpan Zhang [1,2]* and Haodong Tang [1]

1 College of Chemical Engineering, Zhejiang University of Technology, Hangzhou 310014, China; guanjian2020@126.com (J.G.); yuxiaodi@zjut.edu.cn (X.Y.); 15058967256m@sina.cn (M.H.); hanwf@zjut.edu.cn (W.H.); liying@zjut.edu.cn (Y.L.); zjliu@zjut.edu.cn (Z.L.); tanghd@zjut.edu.cn (H.T.)

2 School of Biological and Chemical Engineering, Zhejiang University of Science and Technology, Hangzhou, Zhejiang 310023, China

* Correspondence: zhangpanpan@zjut.edu.cn; Tel.: +86-15868839686

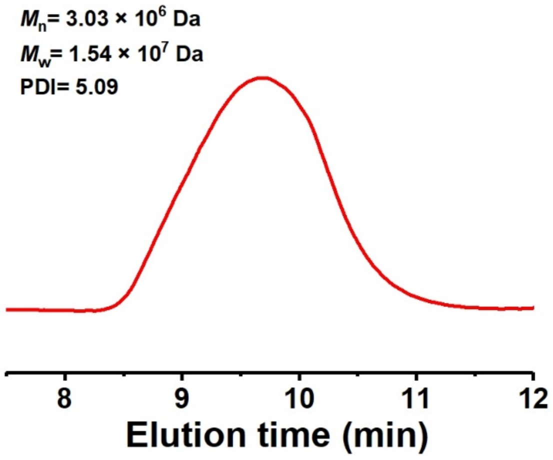

Figure S1. SEC curve of the ultrahigh molecular weight PTFEMA.

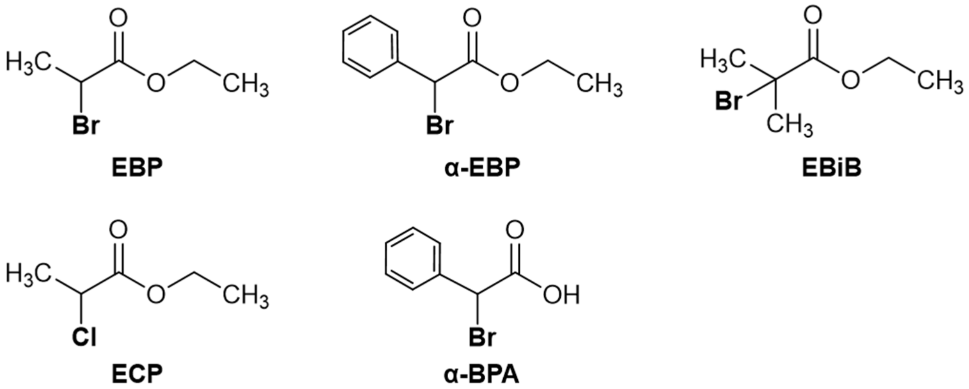

Figure S2. Chemical structures of the substances *α*-EBP, *α*-BPA, ECP, EBiB and EBP (Table 1)
